# Involvement of VDAC, Bax and Ceramides in the Efflux of AIF from Mitochondria during Curcumin-Induced Apoptosis

**DOI:** 10.1371/journal.pone.0006688

**Published:** 2009-08-20

**Authors:** Alwin Scharstuhl, Henricus A. M. Mutsaers, Sebastiaan W. C. Pennings, Frans G. M. Russel, Frank A. D. T. G. Wagener

**Affiliations:** 1 Department of Pharmacology and Toxicology, Radboud University Nijmegen Medical Centre, Nijmegen Centre for Molecular Life Sciences, Nijmegen, The Netherlands; 2 Department of Orthodontics and Craniofacial Development, Radboud University Nijmegen Medical Centre, Nijmegen Centre for Molecular Life Sciences, Nijmegen, The Netherlands; Roswell Park Cancer Institute, United States of America

## Abstract

**Background:**

We previously identified curcumin as a potent inducer of fibroblast apoptosis, which could be used to treat hypertrophic scar formation. Here we investigated the underlying mechanism of this process.

**Principal Findings:**

Curcumin-induced apoptosis could not be blocked by caspase-inhibitors and we could not detect any caspase-3/7 activity. Curcumin predominantly induced mitochondria-mediated ROS formation and stimulated the expression of the redox-sensitive pro-apoptotic factor p53. Inhibition of the pro-apoptotic signaling enzyme glycogen synthase kinase-3β (GSK-3β) blocked curcumin-induced apoptosis. Apoptosis was associated with high molecular weight DNA damage, a possible indicator of apoptosis-inducing factor (AIF) activity. Indeed, curcumin caused nuclear translocation of AIF, which could be blocked by the antioxidant N-acetyl cysteine. We next investigated how AIF is effluxed from mitochondria in more detail. The permeability transition pore complex (PTPC), of which the voltage-dependent anion channel (VDAC) is a component, could be involved since the VDAC-inhibitor DIDS (4,4′-diisothiocyanatostilbene-2,2′-disulfonic acid) efficiently blocked AIF translocation. However, PTPC is not involved in AIF release since cyclosporine A, a specific inhibitor of the complex did not block apoptosis. Alternatively, the pro-apoptotic protein Bax could have formed mitochondrial channels and interacted with VDAC. Curcumin caused mitochondrial translocation of Bax, which was blocked by DIDS, suggesting a Bax-VDAC interaction. Interestingly, ceramide channels can also release apoptogenic factors from mitochondria and we found that addition of ceramide induced caspase-independent apoptosis. Surprisingly, this process could also be blocked by DIDS, suggesting the concerted action of Bax, VDAC and ceramide in the efflux of AIF from the mitochondrion.

**Conclusions:**

Curcumin-induced fibroblast apoptosis is totally caspase-independent and relies on the mitochondrial formation of ROS and the subsequent nuclear translocation of AIF, which is released from a mitochondrial pore that involves VDAC, Bax and possibly ceramides. The composition of the AIF-releasing channel seems to be much more complex than previously thought.

## Introduction

We previously demonstrated that a high dose of curcumin induces apoptosis in human dermal fibroblasts via the formation of reactive oxygen species (ROS) [1]. This apoptotic activity of curcumin is important, since it may be used to diminish scar formation in patients with severe burns. Therefore, we investigated the underlying molecular mechanism in more detail.

Apoptosis, also referred to as programmed cell death, is not only an important mechanism involved in development and homeostatic regulation but also plays a central role in several pathological conditions such as cancer and fibrosis. Generally, apoptosis can occur in either a caspase-dependent or -independent fashion. For caspase-dependent apoptosis two major pathways have been identified: the extrinsic pathway, which involves triggering of death receptors like tumor necrosis factor (TNF) receptor, FAS/CD95-APO-1 and TRAIL/APO-2, and the intrinsic pathway in which the mitochondrion is the central regulator [2]. Fas-associated death domain (FADD) and TNF-receptor-associated death domain (TRADD) recruit and activate caspase-8 to form a death-inducing signaling complex (DISC) [3]. DISC can propagate the death signal generally in two ways. First, DISC can splice Bid, which upon translocation to the mitochondria can cause mitochondrial outer membrane permeabilization (MOMP), leading to the release of pro-apoptotic proteins, such as cytochrome c [4]. Cytochrome c can interact with apoptotic protease activating factor-1 (Apaf-1) and procaspase 9 to form an apoptosome. This complex is able to activate caspase 9, which in turn activates the effector caspases 3, 6 and 7, leading to apoptosis [5]. Alternatively, DISC can also directly activate these effector caspases [6].

The point-of-no-return for the intrinsic pathway is characterized by MOMP, which is controlled by the family of Bcl-2 proteins [7]. These proteins are either anti- or pro-apoptotic and the balance between these two groups ultimately determines cell survival or cell death. The pro-apoptotic Bcl-2 family members Bax and Bak probably contribute to MOMP by interacting with the Permeability Transition Pore Complex (PTPC), which is supposed to consist of the Voltage Dependent Anion Channel (VDAC), Cyclophilin D (CypD) and adenine nucleotide translocase (ANT), and allows for the release of cytochrome c from the mitochondrial intermembrane space [8], [9].

The tumor suppressor p53 is a well known regulator of cell division and apoptosis and curcumin has been shown to induce apoptosis in a p53-dependent fashion [10]. Importantly, one of the mechanisms via which p53 is thought to induce apoptosis is by direct activation of Bax [11]. The serine/threonine kinase glycogen synthase kinase-3 β (GSK-3β) is a multifaceted enzyme that has been shown to interact directly with p53 and to promote p53-dependent apoptosis thus stimulating the mitochondria-mediated intrinsic apoptotic pathway [12], [13]. Consequently, inhibitors of GSK-3β offer protection from intrinsic apoptosis.

The mitochondrial release of apoptogenic factors is an important but poorly understood mechanism. Several of the proteins that are released from the mitochondrial intermembrane space after MOMP have an anti-apoptotic role e.g. HTRA2/Omi and SMAC/Diablo [14], whereas others are pro-apoptotic, such as cytochrome c, apoptosis inducing factor (AIF) and endonuclease G (EndoG) [15]. The release of large apoptogenic factors from the mitochondria remains controversial, since the pore size of PTPC only permits the release of molecules of <13 kDa, like cytochrome c [9]. Alternatively, Bax is known to oligomerize and thus form pores, called mitochondrial apoptosis-induced channel (MAC), in the outer mitochondrial membrane that allows for the release of large (>2 MDa) molecules [16], [17]. Furthermore, it was recently shown that curcumin and other flavonoids induce ceramide synthesis and that ceramides have been implicated in apoptosis via the formation of channels in the outer mitochondrial membrane, which enables the release of large mitochondrial proteins [18]–[20].

Data on the exact mitochondrial localization of AIF are conflicting. Some studies show that soluble AIF is present in the intermembrane space and therefore MOMP alone would be sufficient to release this form of AIF from the mitochondria [21]. Other studies postulate that AIF is inserted in the inner mitochondrial membrane and therefore needs to be cleaved first, by caspases or calpains, followed by MOMP, to efflux from the mitochondrion [22]. Following mitochondrial release, AIF translocates to the nucleus via its nuclear localization signal, where it contributes to chromatin condensation and high molecular weight DNA fragmentation [21]. Furthermore, release of AIF from mitochondria triggers another hallmark of apoptosis, the exposure of phosphatidylserine (PS) on the extracellular side of the cell membrane [21]. The role of EndoG in caspase-independent apoptosis is largely unknown but unlike AIF, translocation of EndoG to the nucleus results in low molecular weight DNA fragmentation, similar to caspase-mediated DNA fragmentation [23].

We have recently demonstrated that curcumin induced ROS-mediated apoptosis in dermal fibroblasts, which could be inhibited by antioxidant treatment or induction of heme oxygenase [1]. Here, we aimed to unravel the mechanism underlying this apoptotic process and we show that curcumin-induced apoptosis depends on mitochondrial ROS-formation and the translocation of AIF from the mitochondrion to the nucleus and is independent of caspase-activity. AIF translocation and subsequent apoptosis could be blocked by N-acetyl cysteine and the VDAC-inhibitor DIDS, but not by the specific PTPC-inhibitor cyclosporin A. Inhibition of VDAC reduced both mitochondrial translocation of Bax and ceramide-induced fibroblast apoptosis. Taken together, our data shows that the release of apoptogenic factors by curcumin from mitochondria involves VDAC, Bax and ceramides, suggesting that the composition of the AIF-releasing channel is possibly much more complex than previously thought.

## Results

### Curcumin-induced apoptosis is not mediated by caspase activity

**Figure 1:**
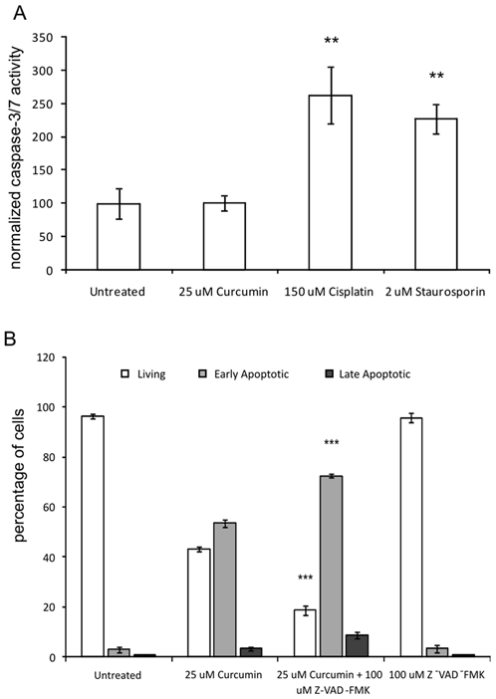
Curcumin-Induced Apoptosis is not Mediated by Caspase Activity. A). Cells were treated for 24 hours with 25 µM curcumin or were left untreated. As a positive control cells were treated with 150 µM cisplatin or 2 µM staurosporin. Caspase-3 and -7 activity was measured using a fluorescence based commercial assay (see M&M). The fluorescence values in the untreated group were set at 100% and the other values were normalized accordingly. Shown are the mean±SD from 3 independent experiments, * = p<0.05 B). Cells were treated with 25 µM curcumin in the presence or absence of the caspase-activity inhibitor z-FAD-fmk (100 µM). Negative controls included untreated cells and cells treated only with z-FAD-fmk. After 24 hours, all cells were collected and stained with Annexin-V-FITC and propidium iodide and quadrant analysis after flow cytometry was performed. Shown are the mean±SD of the living, early and late apoptotic cell fractions from 3 independent experiments, *** = p<0.001 compared to 25 µM curcumin.

We investigated whether curcumin-induced apoptosis was mediated by caspases. Although high levels of pro-caspase-3 were detected by Western blot, we were unable to detect the active form of caspase-3 protein or a reduction in band intensity of pro-caspase-3 after curcumin treatment (results not shown). In addition, curcumin did not induce caspase-3/7 activity in fibroblasts, whereas the apoptosis-inducing agents cisplatin and staurosporin increased caspase-activity significantly (both p<0.01, figure 1A). Moreover, curcumin-induced apoptosis (25 µM) was not blocked by co-incubation with the pan-specific caspase activity inhibitor z-FAD-fmk (figure 1B). There was even a significant decrease in the percentage of living cells, which was accompanied by an increase in early apoptotic cells in the curcumin+z-FAD-fmk group, compared to the curcumin group (p<0.001). Treatment with the caspase-inhibitor alone had no effect on cell viability. Absence of caspase activity was confirmed by the finding that curcumin treatment did not result in internucleosomal low molecular weight DNA damage (DNA laddering, results not shown).

### Curcumin treatment induces Hsp70 expression and translocation

**Figure 2:**
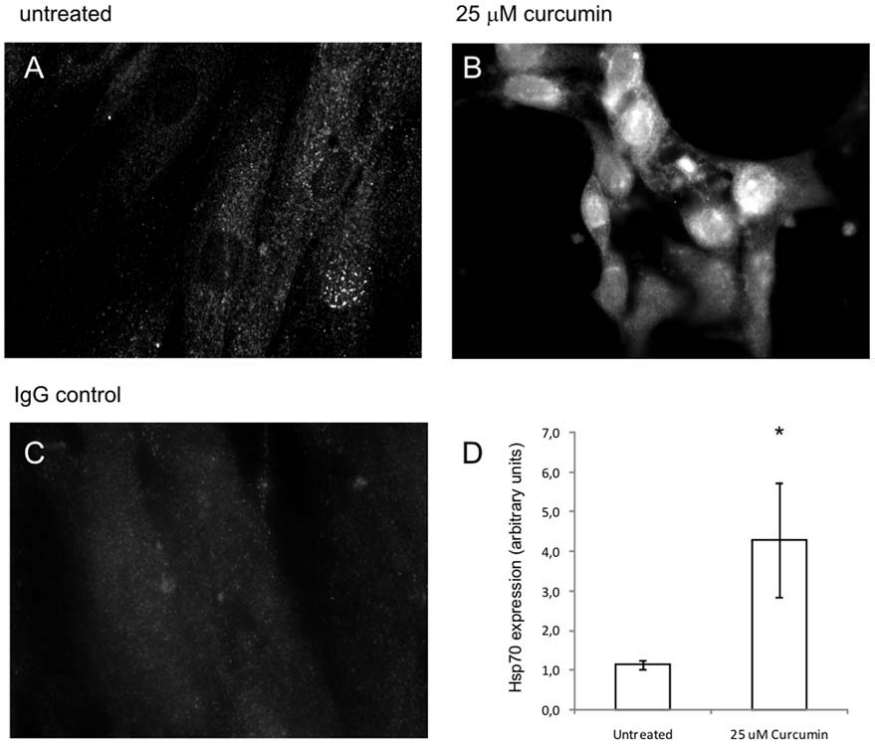
Curcumin Induces Hsp70 Protein Expression. A–C) Fibroblasts were seeded on diagnostic microscope slides and treated with 25 µM curcumin or were left untreated. After 24 hours, cells were fixed and permeabilized and subsequently stained using a specific Ab against Hsp70. Detection was done via an Alexa-488 labeled secondary Ab. As a negative control the primary Ab was replaced with PolyIgG. A) untreated cells, B) 25 µM curcumin, C) Untreated cells IgG control. D) Cells were treated with 25 µM curcumin or were left untreated. After 24 hours cells were lysed and proteins were separated by SDS-PAGE and blotted onto nitrocellulose membranes. Blots were incubated with Abs against Hsp70 and β-actin (as a loading control) and the appropriate fluorescently labeled secondary Abs. Fluorescence of the specific protein bands was determined using the Odyssey Infrared Imaging System. Shown are the mean±SD of the band intensities of Hsp70 corrected for β-actin from 3 independent experiments, * = p<0.05.

Since Hsp70 is an inhibitor of caspase-mediated apoptosis we investigated whether its expression was affected by curcumin treatment. In unstimulated cells Hsp70 was mainly localized in the cytoplasm and some cells showed a spotted pattern in the nucleus (Figure 2A). After 24 hours of curcumin-treatment (25 µM) fibroblasts showed an increase in Hsp70 expression both in the cytoplasm and nucleus (Figure 2B). Quantification of Hsp70 specific bands on Western blot showed that after curcumin treatment expression was ∼4 times higher than in untreated controls (Figure 2D). Thus, curcumin induced the expression of Hsp70, a protein which is possibly involved in the inhibition of caspase activity.

### High molecular weight (HMW) DNA fragmentation

**Figure 3:**
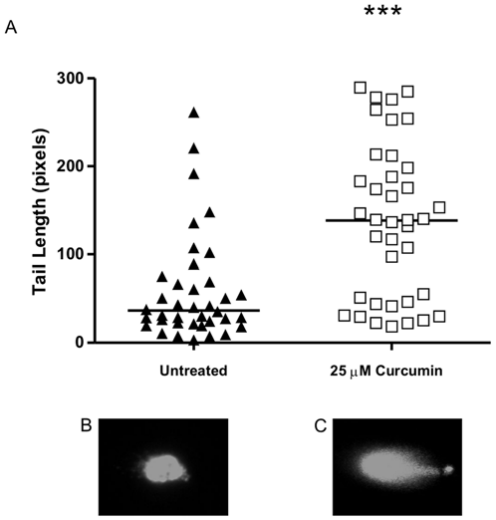
Curcumin Induces HMW DNA Damage. Cells were treated for 72 hours with 25 µM curcumin or were left untreated. HMW DNA damage was studied using the CometAssay Reagent kit. Cells were resuspended in LMAgarose the mixture was placed on to CometSlides. Next, cells were lysed and DNA was released and allowed to unwind. After electrophoresis, slides were fixed and nuclei were stained with SYBR Green. Positive nuclei were analyzed using a fluorescence microscope and a filter for FITC. Nuclei were chosen in a random fashion by a blinded observer using a CCD-camera and a computer monitor. Comet tail length was quantified by a blinded observer using ImageJ image analysis software. A) Shown is a scatter plot were the tail length of the comet of all positive nuclei in the untreated and curcumin-treated group is depicted of 3 independent experiments. The solid horizontal line represents the mean. B) representative picture of a nucleus of an untreated cell. C) representative picture of a nucleus of a curcumin-treated cell, *** = p<0.001.

Besides internucleosomal caspase-mediated DNA damage, HMW DNA damage has also been described during apoptosis. Therefore, we investigated if curcumin treatment resulted in HMW DNA damage via single cell DNA electrophoresis (Comet assay). Figure 3A clearly shows that curcumin treatment resulted in a significantly increased comet tail length, which is a direct measure for HMW DNA damage (average tail length of 59 pixels in the untreated group *vs* 138 pixels in the curcumin-treated group, p<0.001). Figure B and C are representative pictures of the untreated and 25 µM curcumin group, respectively. Curcumin treatment therefore leads to HMW DNA damage in dermal fibroblasts.

### ROS assay

**Figure 4:**
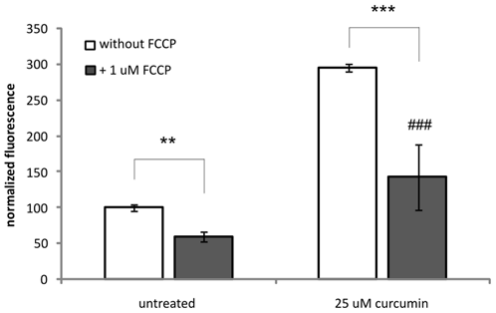
Curcumin-induces ROS formation. Cells were labelled with the ROS-specific labeling dye H_2_DCFDA before they were treated for 45 min with 25 µM curcumin or not treated. To block mitochondrial ROS production the uncoupler agent FCCP was used. After treatment, cells were lysed and fluorescence was determined at excitation 485 nm and emission 520 nm. For each treatment unlabelled cells served as the negative control. Shown are the mean±SD of 3 independent experiments. *** = p<0.001, ** = p<0.01 and ^###^ = p<0.001 compared to untreated - FCCP.

We have previously shown that curcumin induces ROS formation and that antioxidant treatement could fully inhibit curcumin-induced apoptosis [1]. Here, we investigated the cellular origin of ROS formation. Figure 4 shows that 25 µM curcumin significantly induces ROS formation as shown by the increased fluorescent signal compared to untreated cells (p<0.001). After blocking mitochondrial ROS-production by the protonophore FCCP, the fluorescent signal in the curcumin treated cells was significantly lowered (p<0.001), indicating that curcumin-induced ROS formation is predominantly mediated by mitochondria. However, since the signal in the curcumin+FCCP group was still significantly higher than in the untreated situation without FCCP (p<0.001), curcumin also seems to induce ROS formation independent from mitochondria.

### Curcumin induces p53 expression

**Figure 5:**
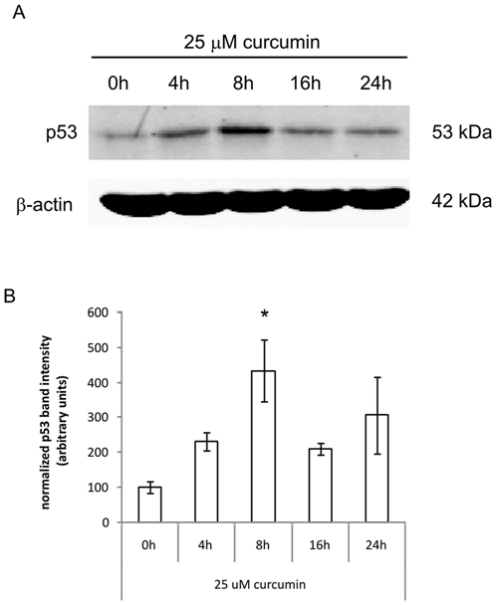
Curcumin induces p53 expression. A). Cells were treated with 25 µM curcumin for the indicated time points before cells were lysed and proteins were separated by SDS-PAGE and blotted onto nitrocellulose membranes. Blots were incubated with Abs against p53 and β-actin (as a loading control) and the appropriate fluorescently labeled secondary Abs. B). Fluorescence of the specific protein bands was determined using the Odyssey Infrared Imaging System. Shown are the mean±SD of the band intensities of Hsp70 corrected for β-actin from 3 independent experiments, * = p<0.05 compared to 0 h.

It is well known that activation of the tumor suppressor p53 can induce apoptosis and therefore we studied whether p53 was involved in curcumin-induced fibroblast apoptosis. Figure 5A+B shows that after 8 hours of curcumin stimulation p53 protein expression was increased 4 fold (p<0.05), compared to unstimulated cells. Already after 4 hours and up to 24 hours following stimulation with curcumin the expression of p53 was elevated, although not statistically significant. Thus, curcumin induces protein expression of the proapoptotic factor p53.

### GSK-3β inhibitors block curcumin-induced apoptosis

**Figure 6:**
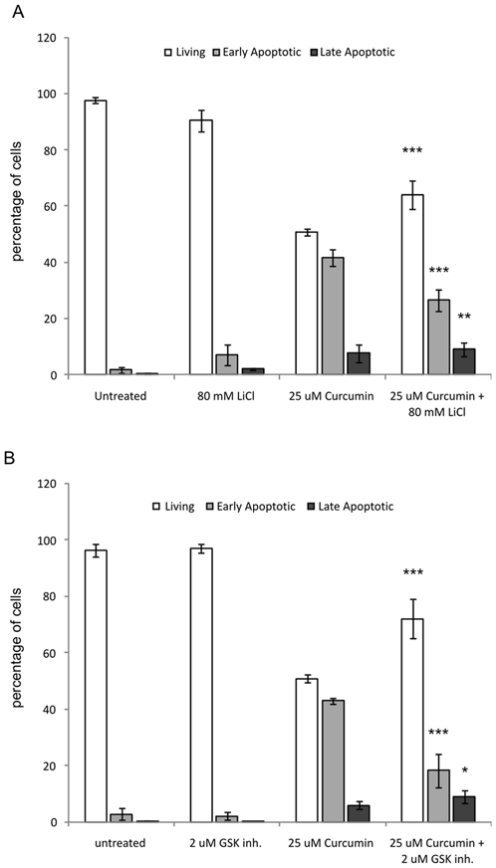
GSK-3β-inhibitors block curcumin-induced apoptosis. Cells were treated with 25 µM curcumin in the presence or absence of the GSK-3β activity inhibitors LiCl (A) or BIO-Acetoxime (B). Negative controls included untreated cells and cells treated only with z-FAD-fmk. After 24 hours, all cells were collected and stained with Annexin-V-FITC and propidium iodide and quadrant analysis after flow cytometry was performed. Shown are the mean±SD of the living, early and late apoptotic cell fractions from 3 independent experiments, *** = p<0.001, ** = p<0.01 and * = p<0.05 compared to 25 µM curcumin.

Inhibitors of GSK-3β can protect cells from apoptosis [13] and it has been shown that p53 is a direct substrate of GSK-3β activity [12]. Therefore, we tested if inhibition of GSK-3β-activity affected curcumin-induced apoptosis. Both pharmacological inhibitors of GSK-3β-activity, lithium and BIO-Acetoxime, were able to significantly increase the percentage of living cells to 65% and 72%, respectively, compared to 51% in curcumin-treated cells (figure 6A+B, both p<0.001). Correspondingly, the percentage of apoptotic cells was significantly decreased to 18% and 27% in the groups were GSK-3β was inhibited, compared to 42% after curcumin treatment only (both p<0.001, figure 6A+B). These results indicated that GSK-3β-activity mediates curcumin-induced apoptosis.

### Curcumin induces translocation of Bax

**Figure 7:**
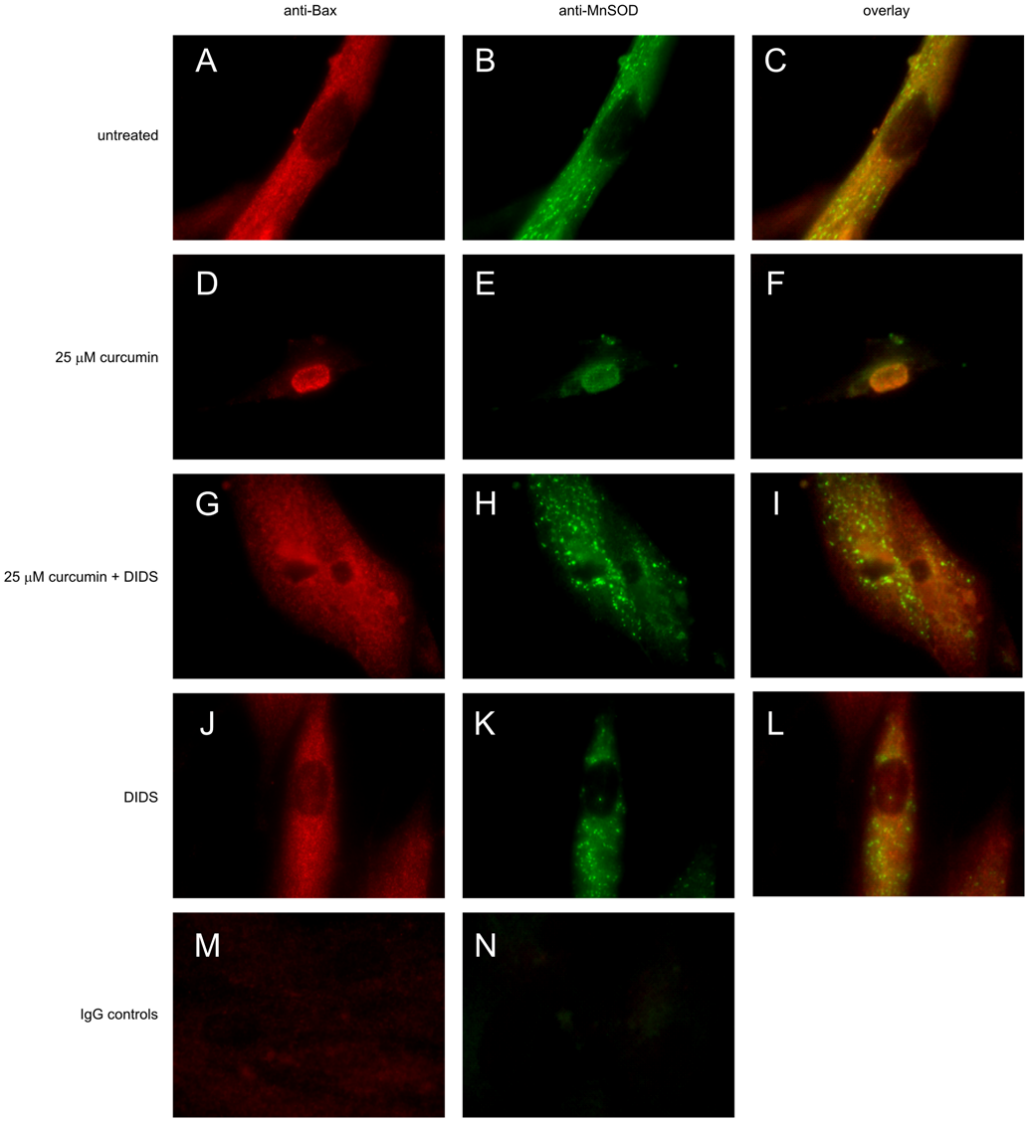
Curcumin Induces Bax Translocation. Fibroblasts were seeded on diagnostic microscope slides and treated with 25 µM curcumin, 500 µM DIDS, the combination, or were left untreated. After 24 hours, cells were fixed and permeabilized and subsequently stained using a specific Ab against Bax A,D,G,J) and the mitochondrial protein MnSOD B,E,H,K). Detection was done via Alexa-488 for MnSOD (green) and Alexa-594 for Bax (red) labeled secondary Ab. C,F,I,L) are overlays of green and red images. A–C) untreated, D–F) 25 µM curcumin, G–I) 25 µM curcumin+500 µM DIDS, J–L) 500 µM DIDS. PolyIgG controls for M) Bax and N) MnSOD.

A well known down-stream target of p53-activity is the pro-apoptogenic factor Bax [11], [24] and, moreover, Bax has been implicated in the formation of mitochondrial channels that are involved in the release of apoptogenic proteins. Therefore, we studied cellular localization of Bax during curcumin-induced apoptosis. Figure 7A–C shows that in the untreated group, Bax has a diffuse cytoplasmatic localization outside the mitochondria since the mitochondria-specific protein MnSOD did not colocalize with Bax. However, after curcumin treatment mitochondria gathered around the nucleus and Bax was translocated to the mitochondria, as indicated by the co-localization with MnSOD (figure 7D–F). This showed that curcumin induced the translocation of the pro-apoptogenic factor Bax to the mitochondria.

### Translocation of Apoptosis Inducing Factor (AIF)

**Figure 8:**
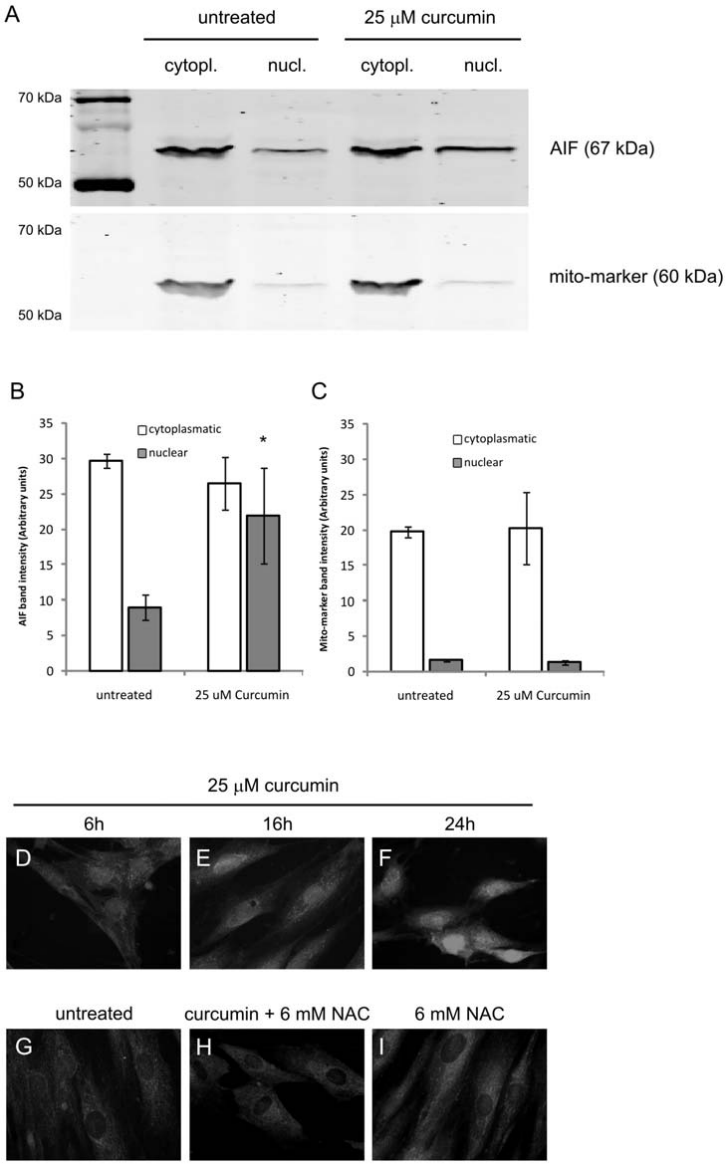
Curcumin Induces AIF Translocation to the Nucleus. A–C) Cells were treated with 25 µM curcumin or were left untreated. After 24 hours cells were lysed and nuclear and cytoplasmatic proteins were isolated using a commercial kit. A) Identical protein concentrations were loaded and separated by SDS-PAGE and blotted onto nitrocellulose membranes. Blots were incubated with Abs against AIF and a mito-marker (as a control for protein separation) and the appropriate fluorescently labeled secondary Abs. The fluorescence of the specific protein bands was determined using the Odyssey Infrared Imaging System. Shown is the result of a representative of 3 independent experiments. B) Mean±SD of the band intensities of AIF from 3 independent experiments. C) Mean±SD of the band intensities of mito-marker from 3 independent experiments. D–I) Fibroblasts were seeded on diagnostic microscope slides and treated with 25 µM curcumin or were left untreated. After 24 hours, cells were fixed and permeabilized and subsequently stained using a specific Ab against AIF. Detection was done via an Alexa-488 labeled secondary Ab. D) 6 hours 25 µM curcumin, E) 16 hours 25 µM curcumin, F) 24 hours 25 µM curcumin, G) untreated cells, H) 24 hours 25 µM curcumin+6 mM NAC, I) 24 hours 6 mM NAC, * = p<0.05 compared to nuclear AIF in the untreated group.

A mechanism to explain HMW DNA damage and phosphatidylserine flip-flop in a caspase-independent fashion is translocation of AIF from mitochondria to the nucleus [21], [25]. Our cellular fractionation studies showed that after curcumin treatment significantly more nuclear AIF was found than in untreated cells (p<0.05, figure 8A–B). A specific mitochondrial protein marker was shown not to be present in the nucleus after curcumin treatment, indicating that the fractionation procedure was not confounded by cross contamination (Figure 8C). Furthermore, our immunocytochemistry results clearly showed that in untreated cells AIF was located in the cytoplasm and not the nucleus (figure 8G), whereas after addition of curcumin a time-dependent increase of AIF expression in the nucleus was observed, to reach maximum levels after 24 hours (figure Figure 8D–F). In line with our previously published results the antioxidant N-acetyl cysteine was able to completely inhibit AIF translocation (Figure 8H) [1]. In conclusion, curcumin causes nuclear translocation of the mitochondrial pro-apoptotic factor AIF.

### Mitochondrial Inner Membrane Potential

**Figure 9:**
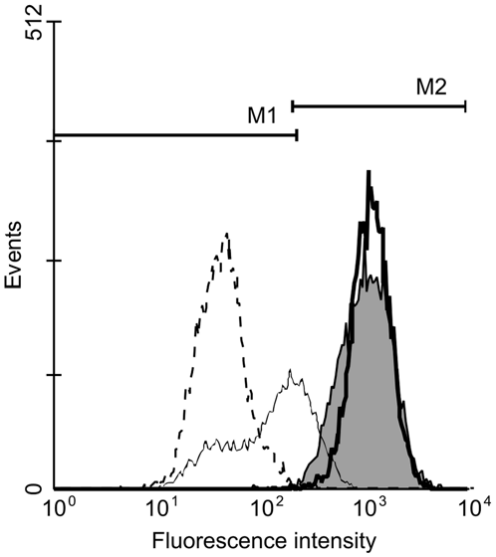
Curcumin does not Cause Mitochondrial Inner Membrane Depolarization. Fibroblasts were treated with 25 µM curcumin or were left untreated. After 24 hours, cells were labeled for 30 minutes with 0.1 µM of the fluorescent dye tetramethylrhodamine ethyl ester (TMRE), which accumulates in mitochondria. As a negative control cells were not labeled. To check if the cells were correctly labeled the protonophore FCCP was added, which causes mitochondrial membrane depolarization. Fluorescence was determined using flow cytometry using channel FL2. Grey peak = untreated cells, thick black line = 25 µM curcumin, thin black line = FCCP treatment of untreated TMRE-labeled cells, dashed line = unlabelled cells. M1 represent negatively labeled cells, M2 represents positively labeled cells.

The release of AIF indicated that curcumin rendered the outer mitochondrial membrane permeable to proteins. We investigated if the permeability of the inner membrane was also affected by quantifying mitochondrial accumulation of the mitochondria-specific dye TMRE, an indicator of membrane potential loss. Figure 9 shows that there was no difference between the accumulation of TMRE in mitochondria of curcumin-treated cells and untreated cells. As a positive control, we confirmed that treatment of TMRE-labeled cells with the mitochondrial protonophore FCCP resulted in a lower fluorescence caused by the depolarization of the membrane potential. In unlabelled cells no fluorescent signal was observed (Figure 9). Therefore, curcumin did not affect the permeability of the inner membrane.

### Inhibition of AIF translocation

Several mechanisms have been described to be involved in the release of AIF from mitochondria. Our results with the pan-specific caspase-inhibitor rule out a role for caspases in AIF release during curcumin-induced fibroblast apoptosis (figure 1B). PARP-activity is also not involved in the release of AIF since co-incubation of curcumin with the specific PARP-inhibitor DPQ did not affect curcumin-induced apoptosis (Table 1).

**Table 1:** Effect of specific inhibitors of various intracellular pathways on curcumin-induced apoptosis.

Treatment a	Living b	Early Apoptotic b
	mean±SD	mean±SD
Untreated	96±2	4±2
25 µM Curcumin	40±9	57±8
25 µM Curcumin+30 µM DPQ c	30±10	66±9
25 µM Curcumin	52±5	41±3
25 µM Curcumin+50 µM NPPB d	32±12**	53±9^*^
25 µM Curcumin	43±4	52±1
25 µM Curcumin+2.5 µM Cyclosporin A e	40±9	56±14
200 µM Cisplatin	28±6	43±7
200 µM Cisplatin+2.5 µM Cyclosporin A e	45±3**	32±2^*^
25 µM Curcumin	38±8	58±5
25 µM Curcumin+5 µM Myriocin f	32±2	65±4
25 µM Curcumin+5 µM Myriocin^f^ (Pre) g	34±4	63±2
25 µM Curcumin	42±14	54±11
25 µM Curcumin+100 µM Fumonisin f	39±15	56±11
25 µM Curcumin+25 µM Fumonisin f (Pre) g	43±25	50±17
25 µM Curcumin	47±13	49±10
25 µM Curcumin+100 µM Manumycin f	47±14	49±11
25 µM Curcumin+100 µM Manumycin f (Pre) g	45±14	51±11

a Cells were treated for 24 hours as indicated and subsequently stained with Annexin-V-FITC and propidium iodide and quadrant analysis after flow cytometry was performed.

b Shown are the mean % ±standard deviation (SD) from the living and early apoptotic cell fractions from 3 independent experiments.

c DPQ was used as an inhibitor of PARP activity.

d NPPB was used as a specific inhibitor of chloride channels.

e Cyclosporin was used as a specific-inhibitor of the PTPC.

f Inhibitors of specific steps in ceramide synthesis.

g “pre” indicates that the inhibitor was given to the cells 24 hours prior to curcumin.

** = p<0.01 and ^*^ = p<0.05.

**Figure 10:**
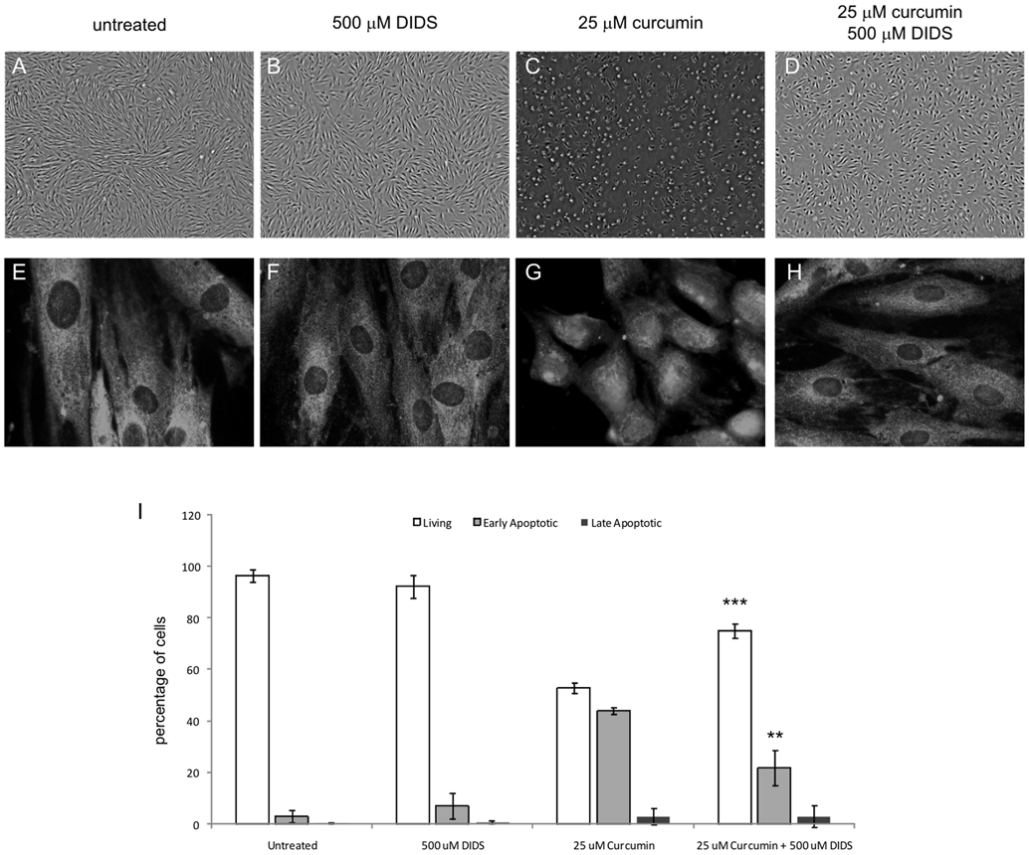
DIDS Inhibits Curcumin-Induced Fibroblast Apoptosis and AIF Translocation to the Nucleus. Cells were seeded on diagnostic microscope slides and treated with 25 µM curcumin, 500 µM DIDS, the combination, or were left untreated. A–D) Cell morphology after 24 hours of treatment. E–H) Immunocytochemistry of AIF. Cells were fixed and permeabilized and subsequently stained using a specific Ab against AIF. Detection was done via an Alexa-488 labeled secondary Ab. A+E) untreated, B+F) 500 µM DIDS, C+G) 25 µM curcumin, D+H) 25 µM curcumin+500 µM DIDS. I) Flow cytometry data. Cells were collected and stained with Annexin-V-FITC and propidium iodide and quadrant analysis after flow cytometry was performed. Shown are the mean±SD from the living, early and late apoptotic cell fractions from 3 independent experiments, *** = p<0.001 and ** = p<0.01 compared to 25 µM curcumin.

The anion exchanger and channel inhibitor DIDS also inhibits the PTPC by blocking VDAC. Figure 10 clearly shows that treatment with 500 µM DIDS potently blocked curcumin-induced apoptosis (Figure 10C+D and I). The percentage of living cells significantly increased from 53% in the curcumin-treated group to 75% in the curcumin+DIDS group (p<0.001), whereas DIDS alone had no significant effect on cell survival (Figure 10B+I). Correspondingly, the early apoptotic cells decreased significantly from 44% in the curcumin group to 22% in the curcumin+DIDS group (P<0.01). Next, we investigated whether DIDS provided protection by abrogation of curcumin-induced nuclear AIF translocation. The data presented in figure 10E–H confirmed that AIF localization in the curcumin+DIDS group was mainly cytoplasmic and not nuclear, which was comparable to the untreated situation.

To test if DIDS acted through inhibition of chloride channels we measured the effect of the specific chloride channel inhibitor NPPB. Interestingly, NPPB did not rescue cells from curcumin-induced apoptosis but rather co-treatment of curcumin with NPPB significantly augmented apoptosis from 52% living cells in the curcumin group to 32% in the combination group (p<0.01, Table 1). These results suggest that chloride channels were not involved in the translocation of AIF during curcumin-induced apoptosis but that a role for the anion channel VDAC could still be possible.

Combined, these data suggest that AIF was released from the mitochondrial intermembrane space via the PTPC. To further test this possibility we co-treated curcumin-stimulated cells with the PTPC inhibitor, cyclosporin A (CsA) [26]. Surprisingly, co-incubation of CsA and curcumin did not result in a decrease of early apoptotic cells compared to curcumin alone, 56% versus 52% respectively, whereas cisplatin-induced apoptosis was significantly inhibited by CsA (Table 1). These results rule out the PTPC as the AIF releasing pore.

Next, we tested if DIDS affected translocation of Bax, since Bax is known to interact with VDAC and create pores in the mitochondrial outer membrane. Indeed, treatment with curcumin in combination with DIDS not only blocked apoptosis and perinuclear migration of mitochondria but also prevented Bax translocation to the mitochondria (Figure 7G–I), whereas DIDS alone had no effect on AIF localization (Figure 7J–L). Consequently, DIDS blocked curcumin-induced apoptosis, inhibited mitochondrial Bax translocation and prevented the release of AIF from mitochondria.

Taken together, our results indicate that Bax interacted with VDAC to generate a pore that is sensitive to DIDS, insensitive to CsA, and permits the efflux of AIF.

### Ceramides

**Figure 11:**
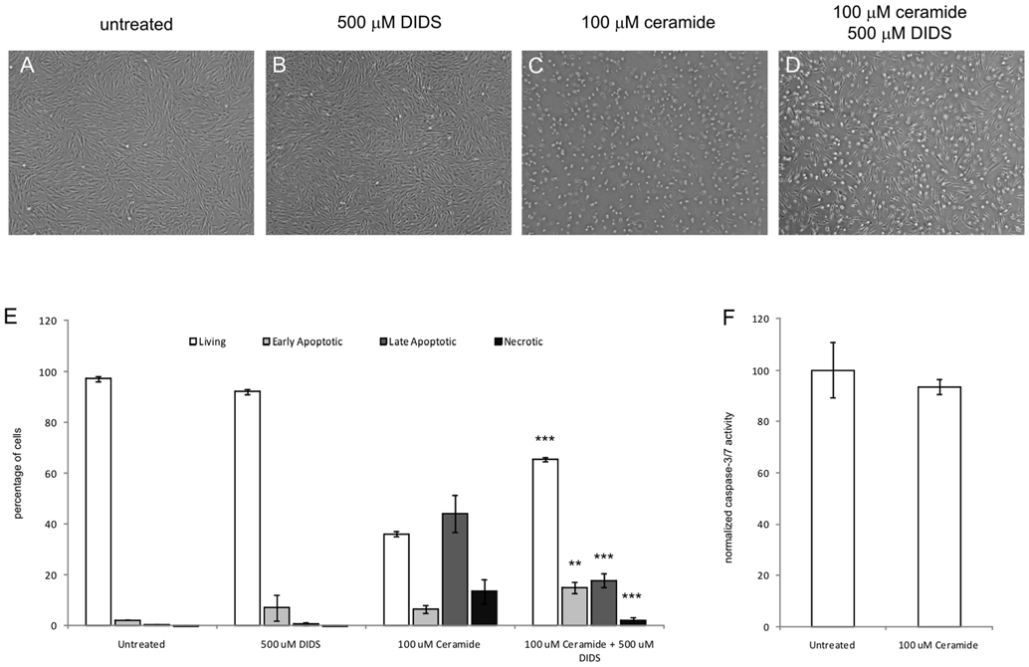
Ceramide Induces Caspase-Independent Fibroblast Apoptosis. Cells were seeded and treated with 100 µM ceramide, 500 µM DIDS, the combination, or were left untreated. A–D) Cell morphology after 24 hours of treatment. E) Flow cytometry data. Cells were collected and stained with Annexin-V-FITC and propidium iodide and quadrant analysis after flow cytometry was performed. Shown are the mean±SD from the living, early and late apoptotic cell fractions from 3 independent experiments. F) Caspase-3 and -7 activity were measured using a fluorescence based commercial assay. The fluorescence values in the untreated group were stated as 100% and the other values were normalized accordingly. Shown are the mean±SD from 3 independent experiments, * = p<0.05 *** = p<0.001 and ** = p<0.01 compared to 100 µM ceramide.

Interestingly, curcumin has been shown to induce ceramide synthesis and ceramides are able to form pores in mitochondrial membranes, thereby allowing for the release of mitochondrial proteins such as AIF. We decreased ceramide levels by inhibition of *de novo* ceramide synthesis via myriocin and fumonisin. However, neither co-incubation of curcumin and the inhibitors nor pretreatment of the cells with inhibitors prior to curcumin treatment resulted in inhibition of curcumin-induced apoptosis (Table 1). Also, blocking the direct formation of ceramide from sphingomyelins by inhibiting sphingomyelinase with manumycin did not rescue fibroblasts from curcumin-induced apoptosis (Table 1). All substances tested were used at a concentration that did not affect cell viability (data not shown). However, we could not exclude the possibility that ceramides present in the mitochondrial membrane were rearranged after treatment with curcumin. Therefore, we studied whether exogenously administered ceramides could induce fibroblast apoptosis. Figure 11A–D shows light microscopic images indicating that 100 µM ceramide caused fibroblast apoptosis and, interestingly, co-treatment with DIDS resulted in protection, as judged from the considerable higher number of attached fibroblast-like cells. Moreover, figure 11E, clearly demonstrates that ceramides significantly decreased the percentage of living cells, which was accompanied by a significant increase in apoptotic cells, compared to untreated and DIDS-treated cells (all p<0.001). Furthermore, co-treatment of ceramides and DIDS significantly improved the percentage of living cells and decreased the percentage of late apoptotic and necrotic cells (all p<0.001). The induction of apoptosis by ceramides was shown to be independent of caspase-activity (Figure 11F). Taken together, we cannot exclude a role for ceramides in the formation of pores, possibly in conjunction with VDAC and Bax, that allow for the release of AIF from mitochondria during curcumin-induced apoptosis.

## Discussion

The mechanism by which curcumin induces apoptosis in non-transformed cells has been linked to both caspase-dependent and –independent pathways. In our model no active caspase-3 or-7 could be detected and the pan-specific caspase-inhibitor z-FAD-fmk did not affect curcumin-induced apoptosis. Piwocka *et al.* previously demonstrated in Jurkat cells that curcumin induced caspase-independent apoptosis, which resulted in HMW DNA damage similar to our data, but mitochondria were not involved. [27], [28]. However, in a later study also in Jurkat cells, the same group described that curcumin can also induce apoptosis in a caspase-dependent fashion [29]. Similarly, curcumin-induced apoptosis in human renal Caki cells has been shown to depend on ROS formation and caspase activity, since antioxidants and caspase inhibitors blocked curcumin-induced DNA fragmentation and apoptosis [30]. Recently, it was found in murine fibroblasts that curcumin induces both caspase-dependent and caspase-independent apoptosis [10]. Similar to our model, in this study curcumin stimulated ROS formation, which led to the release of AIF from the mitochondria. However, curcumin treatment also caused release of cytochrome c and the subsequent activation of caspase-3 and PARP, which could be blocked by the caspase-inhibitor z-FAD-fmk.

In our model no caspase-activity was detected despite high levels of pro-caspase-3 protein in both untreated and curcumin-treated groups (data not shown), suggesting that the caspase-3-activating step was inhibited. Hsp70 is known to inhibit several steps in the caspase-dependent apoptosis pathway including apoptosome formation and the activation of caspase-8, both of which are involved in the activation of caspase-3 [31], [32]. It is tempting to speculate that the increase in Hsp70 protein expression after curcumin treatment is involved in the inhibition of the caspase-dependent pathway in our model, but this needs further investigation. On the other hand, overexpression of Hsp70 has also been described to inhibit AIF and Bax translocation [33], [34]. Obviously, in our apoptosis model the translocation of AIF and Bax was not inhibited by increased Hsp70 expression, probably because in our model the endogenous expression of Hsp70 was increased rather than artificially overexpressed by transduction.

We and others have prevously shown that curcumin induces the production of ROS and that inhibition of ROS generation prevents curcumin-induced apoptosis [1], [10]. Here, we investigated whether the produced ROS were generated in mitochondria or had a cytoplasmatic origin. By using the mitochondrial uncoupler FCCP, which prevents mitochondrial ROS generation, we detected a significantly decreased ROS formation at 25 µM curcumin, indicating that mitochondria are predominantly involved in curcumin-induced ROS formation. These data correspond to previously published results, where lowering the bioenergetic capacity of mitochondria suppressed curcumin-induced cytotoxicity in human cutaneous squamous cell carcinoma cells [35]. However, since the levels of ROS in the curcumin+FCCP group, were still significantly elevated above the level in untreated cells, curcumin also seems to induce mitochondria-independent ROS formation.

Apoptosis is often controlled by the redox-sensitive proapoptotic factor p53 and curcumin is known to induce p53-dependent apoptosis in several cell types [36]. Besides its obvious effects on transcription of numerous proapoptotic factors, p53 is also known to directly activate Bax, which leads to mitochondrial permeabilization and subsequent apoptosis [11]. Our results are in line with these data since we show that in dermal fibroblasts curcumin induces the expression of p53, which is followed by mitochondrial tranlocation of Bax. Possibly, p53 forms a complex with Bax in the mitochondrial membrane as suggested by others [35]. The extend of the non-transcriptional activities of p53 in our model is under current investigation. The pro-apoptotic signaling enzyme GSK-3β is associated with mitochondria-mediated apoptosis since mitochondrial GSK-3β activity is increased during intrinsic apoptosis [36]. Furthermore, it has been shown that GSK-3β is able to bind p53 and that inhibition of GSK-3 activity blocks p53-mediated apoptosis [12]. Indeed, in our apoptosis model inhibition of GSK-3β activity by two structurally different inhibitors, rescued cells from curcumin-induced apoptosis, which is in line with a GSK-3β - p53 interaction. So, curcumin induces p53 expression, which in concert with GSK-3β, and possibly as one large protein complex, causes mitochondrial translocation of Bax.

Although a lot of research on AIF has been performed it still remains unclear if it is cleaved from the inner membrane or present in a soluble form in the intermembrane space. (ADP-ribose) polymerase-1 (PARP) activity has been shown to be an important activator of caspase-independent apoptosis and involved in the release of AIF, which eventually leads to high molecular weight DNA fragmentation, MOMP and the release of cytochrome c [37]. But in our hands inhibition of PARP activity by its specific inhibitor DPQ did not block curcumin-induced apoptosis, indicating that PARP activity did not play a role in the release of AIF. Alternatively, it is often assumed that caspase activity is essential for cleaving AIF from the inner mitochondrial membrane, causing the protein to become soluble in the intermembrane space [38]–[40]. In line with our data, others have reported that in the presence of the pan-specific caspase inhibitor z-FAD-fmk, AIF is still able to translocate to the nucleus, which argues against a role for caspases in the release of AIF [21], [37]. Calpains are also thought to cleave AIF from the inner membrane [22] but since z-FAD-fmk is known to inhibit calpain activity it is likely that in our model curcumin-induced apoptosis relies on soluble AIF present in the mitochondrial intermembrane space.

DIDS is a non-selective inhibitor of anion exchangers and channels and it can prevent apoptosis by a yet unknown mechanism. DIDS has been described to inhibit apoptosis via prevention of cell volume decrease by blocking volume sensitive outwardly rectifying (VSOR) channels. However, in our flow cytometry studies curcumin-treatment did not change the forward scatter of cells, indicating that cell size was unaltered (data not shown). Another likely target for DIDS in the inhibition of apoptosis is VDAC, which is located in the outer mitochondrial membrane. It was recently reported that DIDS prevented cisplatin-induced cell death by inhibiting conformational activation of Bax, which was shown to depend on VDAC [41]. In another recent study, DIDS attenuated staurosporin-induced apoptosis in cardiomyocytes partly by preventing the translocation of Bax, similar to our findings, but in this study apoptosis was mediated by cytochrome c and caspase activity and not by AIF [42]. How DIDS influences the release of mitochondrial proteins requires further investigation.

The release of large apoptogenic proteins from the mitochondrial intermembrane space during apoptosis remains controversial. The PTPC consists of the anion channel VDAC, ANT and CypD and has long been implicated in this process but its pore size is too small to allow passage of AIF [43]. Since we observed no change in membrane potential after curcumin stimulation, ANT, which is located in the inner mitochondrial membrane, could not be involved. Also the potent and specific CypD-inhibitor CsA had no effect on curcumin-induced apoptosis, further ruling out the involvement of the PTPC. The pro-apoptotic protein Bax can interact with VDAC and has been shown to be essential for the release of AIF [8], [22]. However, the pores formed by this interaction are only permeable to proteins of ∼13 kDa like cytochrome c and not the much larger AIF protein (57 kDA) [9]. Bax by itself can also oligomerize and form supramolecular openings that would allow for the release of AIF [17], [44]. The strong rescuing effect of DIDS on curcumin-induced apoptosis as observed in this study, however, makes it hard to envision a channel without a role for VDAC. It is intriguing how DIDS, a drug thought to act on mitochondrial VDAC, prevents the translocation of Bax from cytoplasm to mitochondria. DIDS perhaps binds to VDAC, which is thought to act as an anchor point, in such a way that the interaction with Bax is hindered. On the other hand, inhibition of VDAC by DIDS could block an as yet unknown signal originating from mitochondria, which triggers Bax translocation.

Curcumin and other flavonoids increase ceramide levels and ceramides are known to induce apoptosis [19], [20]. Combined with the fact that ceramides can generate large pores in mitochondrial membranes [45], [46], this led us to investigate whether ceramides could play a role in curcumin-induced apoptosis. Inhibition of *de novo* synthesis and inhibition of the ceramide-generating enzyme sphingomyelinases did not significantly block curcumin-induced apoptosis, indicating that ceramides either are not involved or are sufficiently present in mitochondrial membranes. The addition of exogenous ceramides indeed resulted in fibroblast apoptosis and, strikingly, this form of apoptosis was not only independent of caspase-activity but could also be inhibited by DIDS in a similar fashion as curcumin-induced apoptosis, therefore signifying a role for VDAC in ceramide-induced apoptosis. These data are suggestive for a concerted action of ceramides, VDAC and possibly Bax in the formation of channels in the outer mitochondrial membranes to release AIF.

The mechanism via which curcumin induces apoptosis seems to be complex and dependent on the cell type studied and caspase-activity may be a confounding factor in the identification of the exact mechanism how apoptogenic factors are released from mitochondria. We show in our model that curcumin induces predominantly the formation of mitochondria-mediated ROS. Curcumin-induced apoptosis is totally independent from caspase-activity, and, more specifically, relies on AIF release, since we observed nuclear translocation of AIF and HMW DNA damage. Inhibition of AIF translocation also blocked curcumin-induced apoptosis. In our attempt to identify via which mechanism AIF is released from mitochondria after curcumin stimulation we gathered evidence that not only VDAC and Bax, but also ceramides, are involved in the formation of a channel that permits the release of large proteins such as AIF from the mitochondrial intermembrane space. The unique caspase-independent apoptosis model described here can give important clues to further research on how apoptogenic factors are released from mitochondria.

## Materials and Methods

### Fibroblast culture

Human foreskin-derived fibroblasts were cultured in Dulbecco's modified Eagle's medium (DMEM) with high glucose, containing 10% fetal calf serum (MP Biomedicals, Uden, the Netherlands) and 1% penicillin/streptomycin/amphotericin B (Invitrogen life sciences, Breda, the Netherlands) at 37°C in an 5% CO_2_ atmosphere. Medium was refreshed every 2–3 days and when the cells reached ∼90% confluence they were subcultured using trypsin-EDTA (Invitrogen) at a 1∶3 dilution.

### Western blotting

Cleavage of caspase-3, expression levels of Hsp70, p53 and the subcellular localization of AIF was investigated by Western blotting after 25 µM curcumin treatment and compared to untreated cells. Subcellular localization of AIF was studied using the NE-PER Nuclear and Cytoplasmic Extraction Reagent (Pierce, Thermo Fisher Scientific, Etten-Leur, The Netherlands) according to manufacturers protocol. SDS-Page and Western blotting procedure were essentially performed as described previously [1]. Blots were incubated for 1 hour at RT with mouse anti-caspase-3 Ab (1∶500; eBiosciences, AMDS Benelux, Malden, The Netherlands), rabbit anti-Hsp70 (Koma Biotech Inc. via Abcam), mouse anti-p53 (1∶2500; BD Biosciences, Breda, The Netherlands) or rabbit anti-AIF Ab (1∶1000, Millipore, Amsterdam, The Netherlands). Rabbit-anti-β-actin Ab served as a protein loading control (1∶1000, Abcam, Cambridge, UK). The quality of cell fractionation was verified by staining for a mitochondria-specific protein (1∶500, mouse anti-mito-marker, Abcam). The secondary antibodies used were, goat-anti-mouse Alexa Fluor 680 (1∶20.000, Rockland, Heerhugowaard, the Netherlands) and goat-anti-rabbit IRDye 800 (1∶20.000 Sigma-Aldrich, Zwijndrecht, The Netherlands). The membranes were scanned using the Odyssey Infrared Imaging System (LI-COR Biosciences, Westburg B.V., Leusden, The Netherlands).

### Caspase-3/7 activity assay

Fibroblasts were seeded in a 96-well plate and treated for 48 hours with different doses of curcumin, ceramide or as a positive control with cisplatin and staurosporin, or were left untreated (negative control). Caspase-3/7 activity was determined by the Apo-ONE Caspase-3/7 Assay (Promega, Leiden, The Netherlands) according to the manufacturers recommendations. Formation of the fluorescent product of caspase activity was measured using a Polarstar Galaxy plate reader (BMG LabTechnologies GmbH, Offenburg, Germany).

### Comet assay

HMW DNA damage was studied using the CometAssay Reagent kit (Trevigen, SanBio b.v., Uden, The Netherlands). Fibroblasts were plated at 70% confluence in 6-well plates and allowed to adhere for at least 6 hours before the cells were treated with varying doses of curcumin for 72 hours. Cells were released by trypsin and 10,000 cells were resuspended in 75 µl LMAgarose and subsequently the agarose-cell mixture was placed on to CometSlides. After incubating the slides for 30 min at 4°C to let the agarose solidify, the slides were incubated for 60 min in lysis buffer at 4°C to lyse the cells and release DNA. Next, slides were transferred to a horizontal electrophoresis tray (Owl separation system) filled with an alkaline electrophoresis buffer (300 mM NaOH, 1 mM EDTA, pH>13) and DNA was allowed to unwind during 30 min at 4°C. Electrophoresis was carried out at 25 V for 30 min at 4°C, after which the slides were washed three times with neutralization buffer (0.4 M Tris-HCl, pH 7.5). CometSlides were fixed with 100% ethanol for 10 min, air dried and DNA was visualized using SYBR Green I solution (1∶10,000 dilution in TE buffer, 5 min at 4°C). SYBR Green positive nuclei were analyzed using a fluorescence microscope (Leica DM RA) using a filter for fluorescein isothiocyanate (FITC). Per treatment 38 nuclei were chosen in a random fashion by a blinded observer using a Cohu high-performance charge-coupled device camera and a computer monitor. Comet tail length was quantified by a blinded observer using ImageJ image analysis software (National Institutes of Health, Bethesda, Maryland, USA, http://rsb.info.nih.gov/ij/).

### Measurement of Reactive Oxygen Species (ROS)

ROS formation induced by curcumin was determined as described previously [1]. In brief, the ROS-specific labeling dye 5-(and-6)-chloromethyl-2′,7′-dichlorodihydrofluorescein diacetate, acetyl ester (H_2_DCFDA, Invitrogen) was used to label the cells (10 µM H_2_DCFDA in Hanks' Balanced Salt Solution (HBSS) for 15 min at 37°C). Subsequently, cells were washed once with HBSS and treated for 45 min with 25 µM curcumin or not treated. To study whether curcumin induced cytoplasmatic or mitochondrial ROS production, we used the uncoupler agent carbonyl cyanide-p-trifluoromethoxyphenylhydrazone (FCCP, Invitrogen), which disrupts ATP synthesis and hence mitochondrial ROS production. Cells were pretreated for 10 min with 1 µM FCCP prior to labeling with H_2_DCFDA and also during the labeling and treatment steps 1 µM FCCP was present. Afterwards, cells were washed with HBSS and fluorescence was determined using a Fluostar Galaxy fluorometer at excitation 485 nm and emission 520 nm (BMG Lab Technologies, Offenburg, Germany). For each treatment unlabelled cells served as the negative control.

### Determination of Mitochondrial Membrane Potential

To investigate if curcumin induced loss of inner mitochondrial membrane potential we determined mitochondrial accumulation of the fluorescent dye tetramethylrhodamine ethyl ester (TMRE, Invitrogen) in curcumin-treated and untreated fibroblasts. To check if the cells were correctly labeled the mitochondrial protonophore FCCP (p-trifluoromethoxy carbonyl cyanide phenyl hydrazone, Invitrogen) was added, which induces mitochondrial membrane depolarization. As a negative control, cells were not labeled. After treatment cells were collected and labeled for 30 minutes with 0.1 µM TMRE (Invitrogen). Next, cells were washed with PBS and fluorescence was determined using flow cytometry (FACScan, BD Biosciences) using channel FL2.

### Modulation of curcumin-induced apoptosis

We investigated which factors are involved in curcumin-induced apoptosis. To test whether caspases are involved in mitochondrial release of AIF during curcumin-induced apoptosis, fibroblasts were treated with 25 µM curcumin in the presence or absence of the pan-specific caspase inhibitor benzyloxycarbonyl-Val-Ala-Asp fluoromethylketone (z-VAD-fmk, 100 µM, R&D Systems, Abingdon, UK). LiCl (80 mM, Sigma-Aldrich) and BIO-Acetoxime (2 µM, Calbiochem, San Diego, CA, USA) were used to study their inhibitory effect on GSK-3β-activity. The specific inhibitor DPQ (3,4-Dihydro-5-[4-(1-piperidinyl)butoxyl]-1(2H)-isoquinolinone, Sigma-Aldrich, 30 µM) was used to block PARP-activity. The effect of the anion channel blocker DIDS (4,4′-diisothiocyanatostilbene-2,2′-disulfonic acid disodium salt hydrate, Sigma-Aldrich, 500 µM) and the specific chloride channel inhibitor NPPB (nitro -2 -(4- phenylpropylamino) benzoic acid, Sigma-Aldrich, 50–100 µM) on curcumin-induced apoptosis was also studied. The effect of blocking ceramide generation on curcumin-induced apoptosis was studied by blocking *de novo* synthesis of ceramide by pretreating the cells for 24–48 hours before exposure to curcumin with different doses of myriocin (Sigma-Aldrich, 5–50 µM) or fumonisin (Sigma-Aldrich, 50–100 µM). Direct generation of ceramide from sphingomyelins by sphingomyelinase during curcumin-induced apoptosis was blocked by addition of different doses of manumycin (Sigma-Aldrich, 1–10 µM). Ceramides (Sigma-Aldrich) were added in a concentration of 100 µM to fibroblasts to check if apoptosis was induced in the presence or absence of 500 µM DIDS. Cyclosporin A (CsA, Sigma-Aldrich) in a concentration of 2.5 µM was used as a blocker of the PTPC to test if this channel was involved in curcumin-induced apoptosis. All tested inhibitors were used at a concentration that did not affect cell viability. Apoptosis was investigated by flow cytometry (see below). Fibroblasts were plated at 70% confluence in 6-well plates for FACS analysis or for immunocytochemistry purposes on 12-well diagnostic microscope slides (Cel-Line, Thermo Scientific, Braunschweig, Germany) and allowed to adhere for at least 6 hours before cells were treated.

### Flow cytometry

Flow cytometry analysis was used to differentiate between living, early apoptotic, late apoptotic/necrotic and necrotic cells by staining with Annexin V-FITC and propidium iodide (PI). After 48 hours of treatment, all cells were collected and 100,000 cells were resuspended in 100 µl binding buffer containing Annexin V-FITC and PI according to the manufacturers recommendations (BioVision Inc, ITK Diagnostics b.v., Uithoorn, The Netherlands). Next, samples were incubated for 15 min. at RT in the dark. As a positive control for the Annexin V-FITC and PI staining, fibroblasts fixed with 4% paraformaldehyde and permeabilized by 0.1% saponine were used. Quantification of Annexin V-FITC and PI binding was performed by a FACScan (BD Biosciences) using channels FL-1 (Annexin V-FITC) and FL-3 (PI). Cellquest Pro was used to perform quadrant analysis.

### Immunocytochemistry

To study cellular localization of AIF, Hsp70, manganese superoxide dismutase (MnSOD, mitochondrial marker) and Bax we used immunocytochemistry. After 24 hours of treatment, cells were washed with PBS and fixed for 15 min in 4% paraformaldehyde in PBS. Next, the cells were washed three times and cells were permeabilized and aspecific epitopes were blocked for 30 min at RT with PBA (1% bovine serum albumin, 0.1% saponin, 0.05% NaN_3_ in PBS) together with 2% serum of the animal species in which the secondary antibody was generated. Primary Ab's rabbit anti-AIF, rabbit anti-Hsp70, rabbit anti-MnSOD (Stressgen, via ITK Diagnostics b.v.) and mouse anti-Bax (Abcam) were incubated for 45 min in PBA at a concentration of 5 µg/ml or as indicated by the manufacturer. As a negative control the primary Ab was replaced by polyIgG at 5 µg/ml. After washing three times with PBA, cells were incubated for 30 min with the appropriate fluorescently labeled secondary Ab (Alexa 594 or Alexa 488) (Invitrogen, 1∶1000 in PBA). Cells were washed twice with PBA and once with PBS before mounting the slides with Fluorescent Mounting Medium (DakoCytomation, Dako Netherlands b.v., Heverlee, Belgium). Fluorescence was studied using a fluorescence microscope (Leica DM RA) using a filter for tetramethyl rhodamine iso-thiocyanate (TRITC) and/or FITC. Images were taken via a Cohu high-performance charge-coupled device camera and a computer monitor.

### Statistical analysis

Statistics were performed using GraphPad Prism 4.03 software via a One-way analysis of variance (ANOVA) followed by the Newman-Keuls multiple comparison test when comparing 3 or more groups. A Student's T-test was used when comparing 2 groups. Differences between groups were stated to be statistically significant when p<0.05. All experiments were repeated at least 3 times.
